# Managing smaller but flexible automation: back to the future

**DOI:** 10.1155/S1463924603000130

**Published:** 2003

**Authors:** Derek J. Hook, David M. Evans, Mark Deiparine, Joseph Russo, Christopher Drabik

**Affiliations:** Psychiatric Genomics Inc. 19 Firstfield Road Gaithersburg MD 20878 USA


Journal of Automated Methods & Management in Chemistry
Vol. 25, No. 3 (May–June 2003) pp. 73–77

Taylor & Francis

Taylor & Francis Group

### Managing smaller but flexible automation: back to the future†

**Derek J. Hook*, David M. Evans, Mark Deiparine, Joseph Russo and Christopher Drabik**

*Psychiatric Genomics, Inc., 19 Firstfield Road, Gaithersburg, MD 20878, USA*

### Development of automation for drug discovery

Practical robotics tools were introduced to the analytical chemistry laboratory about 20 years ago with the development of the Zymate I robot by Zymark Corporation (Hopkington, MA, USA). These tools quickly migrated within the various parts of the research divisions into biology and biochemistry laboratories at pharmaceutical companies to support the new discipline of high-throughput random screening. These systems were small scale and often reconfigured for new screens because of small numbers of compounds in collections at that time.

Over the last 10 years, however, these systems have matured, and it became clear that laboratory automation was becoming a reliable tool for drug discovery. Over this period, management in the pharmaceutical research industry was convinced that this technology and approach was a productivity enhancing tool and invested heavily in infrastructure and people to implement it. During this time, three other trends contributed to the momentum towards large dedicated 'industrialized' screening operations. One was the proliferation of molecular targets that were being produced by the genomics revolution; the second the move towards detection technologies that were either homogeneous or semi-homogeneous eliminating many of the separation steps in assays; and the third the development of large combinatorial chemistry/parallel synthesis capability in the research organizations, often linked to very large-scale compound collections and compound management systems.

The result has been the move towards assembly line-like automation (e.g. Allegro™ and other conveyer-based systems) and the move towards ultra-miniaturization and ultrahigh throughput (e.g. Evotec and Aurora). As a result, the use of workstation and smaller flexible automation approaches has largely remained localized within biotechnology companies or therapeutic biology units with the larger pharmaceutical organizations where large support groups or capital to support these large dedicated automation systems do not exist.

*To whom correspondence should be addressed.
e-mail: dhook@psygenomics.com
†This paper was initially presented at the ISLAR 2002 Conference and is reproduced here by kind permission of Zymark Corporation.

### Decline in approval in new chemical entities as drugs

However, despite trends to make chemistry in lead optimization more efficient ('kill early and kill often'), it is clear from a number of seminal presentations over the last year or so [1, 2] that the promise of industrialized HTS has not been fulfilled, and that the numbers of new chemical entities being approved (NCEs) has declined over the last few years, rather than increased. This is true despite the increased R&D investment in research and in particular in HTS drug-discovery groups.

These same analysts are now reassessing whether or not continued investment in traditional HTS can bring about the revolution in NCE discovery that had been promised from this investment in resources [3].

What are the explanations for this dearth of NCEs? A number of suggestions have been put forward (including the suggestion that we have not had time for the new drugs from the HTS revolution to make it through the regulatory pipeline), but the most likely reason is that the drug-discovery paradigm has been transformed. It is increasingly being focused on large numbers of single molecular targets. Also the 'industrialization' of HTS has contributed to the momentum towards screening targets that are easy to pursue rather than ones that are the most biologically relevant.

For example, this reliance on single, specific molecular targets, such as GPCR subtypes, yield very highly potent and specific compounds, but they do not provide the desired physiological result *in vivo*. This is especially true in the central nervous system (CNS), where GPCRs that have been targeted by very highly specific and potent compounds *in vitro*, but these compounds have turned out to be ineffective in man. Aripiprazole is one example of a recently approved CNS active compounds that is effective *in vivo*, and is one that shows mixed receptor activity. The use of mixed serotonin/norepinephrine reuptake inhibitors are other examples of drugs that are more effective than compounds that target either of the individual reuptake system alone.

Another probable reason is that early combinatorial chemistry programs produced large numbers of compounds that could be easily made rather than the effort being used to generate 'druggable' molecules, i.e quantity over quality. The trend is now towards more intelligent library design, but even now parallel synthesis is still producing compounds that have inherent flaws in ADMET parameters such as poor solubility, poor absorption, drug–drug interactions and susceptibility to first-pass metabolism. *In vitro* and *in silico* tools to 'fail fast' compounds from parallel synthesis programs are being applied but they need to move towards the application of these methods in the design of primary screening

*Journal of Automated Methods & Management in Chemistry* ISSN 1463–9246 print/ISSN 1464–5068 online © 2003 Taylor & Francis Ltd
http://www.tandf.co.uk/journals
DOI: 10.1080/1463924032000121165

libraries, in addition to their application to lead optimization stages.

A third likely reason is the momentum towards the use of mix and measure primary screening assays. This has lead to the use of targets that can be easily established, rather than a consideration of whether these are relevant targets. Although many of them are, in fact, specific single 'druggable' molecular targets, they are the 'low hanging fruit' of target classes. Over the last 10 years, this 'fruit' has been extensively harvested (e.g. GPCRs, kinases, proteases, ion channels), and whilst examples of these still remain as potential targets, most have been extensively exploited. These classes also represent some of the last examples of therapeutic intervention at targets where there can be seen to be a linkage between cause and effect. However, this target focus is rapidly reaching the point of diminishing returns as it is understood that many of the remaining orphan receptors are involved in complex interactions.

In addition, whilst it is possible to screen in cell-based systems using the 'industrialized' approach, this is not typically done using these ultrahigh-throughput automated approaches.

### Looking towards the future: high information-content screens

Collectively, then, this move towards large-scale and ultrahigh throughput has been facilitated by the availability of cloned single molecular targets from the genomics revolution, the explosion in compounds available for screening through automated combinatorial and parallel chemical synthesis and the commercialization of generic homogeneous and semi-homogeneous mix and measure reagents and detection technologies.

However, the diseases that have the most unmet medical need are those that are the most complex (e.g. diabetes, obesity, mental illness). These complex diseases are the result of interactions of many different regulatory and feedback systems that translate the effects of multiple genes on an individual's susceptibility to a disease, its severity and progression with environmental influences and triggers. If we consider a systems biology approach, then it is likely that new treatments will come from a polyvalent effect of new drugs, i.e. an effect in which a single compound affects multiple cellular processes, yielding the desired therapeutic effect. It is also likely that the targets for such compounds are more likely to be intracellular signalling nodes in pathways that act on gene expression patterns that influence the downstream processes. This would also help to explain the delayed onset of action of many CNS agents, where one can imagine that gene expression changes are followed by modulation of protein expression as the ultimate cellular event, rather than the immediate effect of these compounds on cell surface receptors. In order ultimately to understand these diseases, it will be necessary to unravel and integrate the systems biology involved. However, from a drug-discovery perspective, the important message to be taken from this concept is that a systems biology approach to screening is needed, and that this holds better promise for discovery of truly novel therapeutics for treatment of these complex disorders. Often these diseases are currently being treated with poly-pharmacology, e.g. in mental illness or in obesity, and a paradigm shift toward the use of polyvalent drugs may be a solution to this problem of using multiple drugs in a poly-pharmacologic approach.

All this suggests that an approach needs to be developed that is both novel and, for example, directed towards measuring the effect of gene expression when searching for novel therapeutics. This is the direction that we are pursuing at Psychiatric Genomics, Inc. We are are also tackling this problem by using *in vitro* cell-based screening systems rather than isolated biochemical targets. These types of screens measure many parameters simultaneously, and thus they will be very information rich with the potential for yielding both better information on the effects of compounds on multiple targets simultaneously and have the potential for the discover of novel therapeutics more rapidly.

### Drug discovery using the Multi-Parameter High Throughput Screen (MPHTS$^{SM}$)

As indicated above, Psychiatric Genomics is using gene expression assays to approach the complex psychiatric diseases of bipolar, schizophrenia and depression. The platform technology being used relies on measuring the mRNA expression levels of 16 genes simultaneously in a single well of a microplate after cells have been treated with test compounds from a screening library. The results obtained contribute a pattern of readout of gene expression that can be considered as a signature for that particular compound in the particular assay.

To develop gene expression signatures for the screens based on this platform, we obtain the screening signature by combining information from three complementary approaches coupled with a critical biostatistical analysis of gene expression data. For psychiatric diseases, the first approach uses human post mortem brain tissue (specifically quality controlled for PMI, state on death, age matching, medical diagnosis, RNA quality) to measure gene expression patterns in normal versus diseased individuals (bipolar, schizophrenia, depression). We then extract the differential pattern of gene expression and validate this statistically by using signatures from populations of individuals. The relevant changes are then validated by RT-PCR. The second approach uses the gene signature obtained in human neuronal cells treated with therapeutic agents *in vitro* compared with untreated cells. Again a differential gene expression signature is obtained, and again is validated by RT-PCR. The third approach uses the *in vivo* treatment of animals with therapeutic agents and obtains a differential gene expression signature in different animal brain areas between control and treated animals. These three differential gene expression signatures are compared and combined with a biochemical pathway analysis results in the selection of genes for the MPHTS$^{SM}$ array.

### Implementation of the assay

The multiparameter approach that Psychiatric Genomics is using is high in information content, but is also very complex to implement. A description of the assay protocol will been given in detail in another paper at this conference [4], but to summarize, cells are treated for 24 h with test compound in a standard tissue culture microplate and then after incubation under standard conditions are lysed with a buffer containing nuclease protection probes. These DNA probes then hybridize with the mRNAs of interest. The solution is then treated with S1 nuclease to remove all single-stranded nucleic acids, but leaves the mRNA sequences and DNA protection probes protected in this double-stranded format. A subsequent alkaline treatment then degrades the mRNA and leaves the DNA probes both intact and matching the quantity of the original mRNA in the cell lysates. Excess non-duplexed probe has been destroyed in the nuclease treatment. The detection plates are prepared separately. These plates contain the 16-gene probe mini-arrays at the bottom of the wells of a special 96-well microplate. They are treated with sandwich nucleotide detection sequences, and the detection sandwich built up on each of the 16 spots in the well. Subsequently, an aliquot of the lysed cell extract containing the nuclease protected mRNA double-stranded hybrids is added to the detection plates, heated to allow the melting of the protection duplex and cooled to allow subsequent hybridization to the capture probes. Following washing, an additional step hybridizes an enzyme-labelled detection probe to the sandwich, followed by the generation of a chemiluminesce signal by addition of substrate, which is read with a cooled CCD camera.

### Learning from the past (back to the future!)

The successful management of the automation for such an assay in a biotechnology company environment has resulted in a return to the flexible philosophy of laboratory automation that had been practised in the early days of the implementation of robotics for high-throughput screening.

In these early days of laboratory automation, the accessories for the various unit operations of an assay were often not available and had to be built from scratch. In addition, it became clear that certain unit operations were not robot 'friendly' and that the goal of achieving complete assay automation was either impossible or meant adjusting the assay procedure to a new protocol with consequent revalidation of the assay. Nevertheless, using a combination of semibatch automation and manual intervention satisfactory progress towards high-throughput screening goals was achieved. This type of semibatch automation became anathema to many of the practitioners of industrialized automation, and thus fell out of favour. However, what was true then is true now when we want to implement these novel high-information content screens with a reduced but more focused and smaller compound libraries.

Then, as now, since throughputs in high-throughput screening groups were not large, and often because compound collections even at major pharmaceutical companies were small, historically this frequently meant that the robot systems were continually being reconfigured as assays came and went during the attrition process in the screening group, and required a cadre of people with special talents who could cope with the need for systems that required this constant attention.

An example of the semibatch type of operation was used in early cell-based assays, such as the cytotoxicity screens set up in the early days of automation by my (then) group at Bristol-Myers Squibb. In this system, additions of test compounds to the microplates containing the growing cells were automated using a large tracked system containing staging $CO_2$ incubators. The overnight growth was performed off-line by manual removal of the plates from the staging incubators, and the plates with cells returned to the system for processing for measurement of cell viability after 24 or 48 h growth, again this return process being a manual step. This type of semibatch operation is especially well suited to high-volume cell-based screens, where it is undesirable to tie up limited robotic resources just for storage and incubation for long periods. The throughput of cell-based assays often is lower than traditional homogeneous single molecular target assays. However, when optimized, throughputs can still be quite considerable and adequate for high-priority important targets.

We had many of the same challenges automating this assay as I did with these previous cell-based assays. The need to maintain sterility is important during the growth phase of the cells, but the current assay has the complication that we cannot use antibiotics to maintain sterility, because of the effects that exposure to these compounds has on gene expression. This means that special precautions and process operations are needed to ensure sterile operation. In addition, the times involved in the various steps vary widely, the basic manipulations are more complex than are usual (i.e. there are a number of high temperature nucleic acid hybridizations steps involved, plus associated wash steps, followed by addition of chemiluminescence reagents and a time-dependant light signal readout) and commercial high-temperature ovens that were compatible with automation were not available.

Despite these challenges, and following their solution by means outlined below, the final screening throughput is comparable with some other complex cell-based assays such as the intracellular measurement of calcium using fluorescent dyes and the FLIPR™ (Molecular Devices, Sunnyvale, CA, USA). At NPS Pharmaceuticals (Salt Lake City, UT, USA), for example, my (then) screening group routinely ran 65 96-well plates/day in an entirely manual mode, assisted by some liquid-handling workstations for sample preparation. Once fully operating, our multiplexed gene expression assay should reach comparable throughput, and thus would be adequate to screen rapidly quite large-sized compound collections at about 32 000 compounds/month.

### Management decisions based on past experience (back to the future – part II!)

We required a system that was both state-of-the-art and able to be configured to work in a semi-automated fashion. Since we were using a cell-based assay with a minimicroarray-based detection system that required quite long incubation times, this was incompatible with the utilization of valuable robotic resources for the cell growth steps. In addition, just as in the early days, the need to use the system for both assay development and walk up utilization of the peripherals for other non-array-based assays (with a limited budget) precluded complete duplication of the peripherals on the robotic system. We thus needed to include multiuse into the design of the system.

As described, this is not the typical mix and read homogeneous reaction that can be easily automated at ultrahigh throughput! So careful management of the decisions about what sort of automation to employ was essential to establishing a successful high-throughput screening paradigm.

One of the initial choices to be made was whether to take a workstation, dedicated automation or integrated, yet flexible automation approach. The decision to go with a integrated, flexible robotic system based on a CRS tracked robotic arm was partly dictated by the fact that whilst we were in the process of establishing automation for other internal screens (GPCRs) we did not know the exact assay protocol for the MPHTS$^{SM}$ system. Thus, we chose the CRS system based on our ability to use it as an integrated robotic platform, but also viewed it as an integrated single workstation as necessary. It also had the advantage that our automation support personnel had extensive experience with the system and was able to use the scheduler later to assist in workflow optimization. The choice of peripherals was also based on the need to use these on an open access basis during assay development for the MPHTS$^{SM}$ as well as our other single target GPCR assays.

Managing the automation not only has required us to examine carefully the workflows through the system to optimize throughput, but also has required us to integrate the automation of a CCD camera system originally designed for one-shot manual operation. Fortunately, we had the staff able to design and construct a custom automated solution to feed plates from the robot to the camera. Also, the judicious use of a summer intern programme allowed us to develop software that allowed for automated image capture and analysis has allowed us remove this bottleneck to the HTS process.

These two approaches to developing solutions is again reminiscent of the early days of high-throughput automated system development that allowed a system to evolve from prototype to robust implementation through skilled system design and construction.

My experience with other automated cell-based systems at Bristol-Myers Squibb and at NPS Pharmaceuticals ensured that we focused on the integration of other workflow processes to ensured that the system works without bottlenecks. These included the development of a robust system for routinely producing cells in the correct physiological stage for the assay in large quantities, the compound and plate management tools for maximizing the hit rate and retest process, and the development of advanced data analysis methods to handle the large quantities of multiparametric data produced by this high information content screen.

### Results and data processing

The results returned from this type of screen are a very complex and information-rich data set. The system is designed to screen compounds that affect a multiplexed gene expression pattern in a highly parallel fashion, and its early implementation with a six-gene assay has identified compounds that mimic the gene expression effect of a marketed bipolar therapeutic. It has demanded that we not only use an industrial-strength database to store the results (ActivityBase, IDBS, Guildford, UK), but also even with this database the challenges in constructing templates to upload and analyse data from a complex multiparameter screen are enormous, since the database is essentially designed to handle univariate data. This has meant that we have had to develop and implement tools that can analyse data in a multidimensional result space.

Of the multiple gene probes in the mini-arrays in the bottom of each microplate well, some genes are 'test' genes and reveal the effect of the compound on the up or down regulation (or no effect on regulation) of that particular gene. Since we have started with a profile of gene expression levels as the target cut-off in the assay, we can easily identify compounds that meet the criteria for the signature. In addition, we can mine the data set for gene expression pattern changes that can yield compounds that can be useful both as tools to explore the biochemistry of these events, or as a drug for a disease where a compound producing a different signature is desired. As we continue to add MPHTS$^{SM}$ screens to our portfolio, the data warehouse being build rapidly builds in value and can be mined for future signature targets without having to screen physically the entire compound deck.

### Ongoing assay optimization and management

There are tradeoffs to be made using this technology. The cost per assay plate is high compared with assays in traditional format, but the cost per datum point is not inconsistent with other complex HTS being run using expensive, rare reagents. However, experience suggests that both volume usage and incremental improvements in the technology will drive costs down significantly. This has been our experience in the past as any pioneering assay technology becomes more mature.

Meanwhile, measures can be taken to improve the cost/benefit of the MPHTS$^{SM}$. One is to use the power of chemo-informatics to prioritize plate management through the screen to maximize SAR information from primary screening. We have seen an example of how this might be done, using the earlier six-gene version of the MPHTS$^{SM}$ system. We were able to do a similarity search on a standard of known molecular structure used in the assay validation to prioritize a small set of compounds plates to be screened first in the assay. From two 'hits' obtained from one of these plates, we prioritized an additional set of compound library plates based on a refined structure similarity match and increased the 'hit' rate threefold. One continuing challenge for us is to implement the necessary compound management resources to utilize this approach fully. With more effective compound management systems, other people have used *in silico* techniques to cluster screening compounds, to select a subset of the compounds as being representative of the clusters, re-plate and reformat these into smaller screening sets. Follow up of 'hits' by retesting nearest neighbours resulted in an increased hit rate in this secondary round. Frank Brown at Merck [5] has shown that with this approach one need only screen about 20% of a collection to get about 95% of the 'hits'. This can provide early entry to chemistry even if ultimately the entire compound collection is screened, but allows the option to reduce screening costs by 75% if needed.

Much of the ongoing management of the automation process is focused on three areas. The first is acute and chronic risk management. This is focusing on which part of the process is so critical that failure of the equipment would lead to either short- or long-term downtime for the assay, and the back up procedures to minimize both of these effects. It also includes an analysis of how to process samples to minimize the effect of system failure on individual sample batches on both a short- and long-term basis. The second area is focus on throughput optimization. This is directed toward an analysis of the use of either workflow processes or equipment additions to increase the throughput of the assay. One area of attention here is the complexity and amount of data being generated and how to improve both data processing and the use of novel data analysis and visualization techniques to aid chemistry follow up of 'hits'. The third is not related to automation but to two critical parts of the protocol, i.e. tissue culture and assay step optimization. One of the lessons well learned over the years is that the automation process is not necessarily the weakest link in the chain, but that control of cell growth state is critical to ensure a reproducible supply of cells at a particular stage of biological responsiveness and that careful optimization of each stage of an assay process to ensure that each assay step is not balanced at a knife point of response condition to allow some window of process condition which does not result in great variations in output of the process stage.

The measurement of mRNA expression of multiple genes in a cell-based assay, with multiple process steps for expression level detection, makes this a very difficult assay to develop into an industrialized high-throughput screen. My previous experience with the measurement of intracellular calcium changes in HEK-293 cells transformed with recombinant mGluRs using the FLIPR assay at NPS Pharmaceuticals indicated that the state of cell physiology of the recombinant cells was very important for receptor expression and physiological response of the cells. This multiplexed gene expression assay multiplies these considerations many fold. Critical for success in our current assay has been the development of robust QC procedures for measurement of the state of cell physiology both prior to treatment of the cells with compound and also as a QC check for a decision point to commit to the use of the cell lysates in the detection part of the assay.

### Summary

We believe that this approach to high-throughput screening not only will yield more effective and novel therapies for psychiatric disease, but also will do it in a more effective and rapid manner, one that will overtake traditional single-target HTS approaches.

The system that we have put together based on experience in developing flexible, small-scale and reconfigurable automation shows that the future of high-content cell-based assays can be achieved with limited resources and without the need for large-scale 'industrial' automation and suggests that the future of drug discovery lies less with huge, inflexible 'industrialized' systems than more nimble flexible automation. Just as the PC destroyed the mainframe, and just as the Internet destroyed point-to-point telecommunications, will flexible automation destroy industrialized high-throughput screening or will they coexist as mainframes as the telephone coexists with PCs and the Internet?

However, the results achieved to date with our 6-gene MPHTS$^{SM}$ system suggest that a multiparametric approach to drug discovery has the potential to be a more effective approach to the discovery of therapeutic agents acting at unique sites and with novel mechanisms of action.

### Acknowledgements

The array plate kits (microplates with the mini-arrays and probe reagents) were supplied by High Throughput Genomics, Inc., Tucson, AZ, USA, and their support helped develop the assay. Also the authors acknowledge the support of the tissue culture and screening group at Psychiatric Genomics, especially Dr Ali Mohamed and Gloria Hwang, and to Dr Les Klimczak for assistance in developing the data mining techniques, and to Dan Wilcox, our summer intern, who helped develop the automation for the CCD camera and image processing.

### References

1. Screentech 2002 IBC Conference, San Diego, CA, March 2002.
2. Post, G., Plenary session, Drug Discovery Technology meeting, IBC Conferences Boston, MA, August 4–10 2002.
3. Fox, S., Wang, H., Sopchak, L. and Farr-Jones, S. *J. Biomolecular Screening*, **7** (2002), 313.
4. Evans, D. E. *et al.*, ISLAR Proceedings 2002.
5. Brown, F., Scitegic Users Group meeting, San Diego, CA, January 2002.

